# Reliability Study of Solder Paste Alloy for the Improvement of Solder Joint at Surface Mount Fine-Pitch Components

**DOI:** 10.3390/ma7127706

**Published:** 2014-12-02

**Authors:** Mohd Nizam Ab. Rahman, Noor Suhana Mohd Zubir, Raden Achmad Chairdino Leuveano, Jaharah A. Ghani, Wan Mohd Faizal Wan Mahmood

**Affiliations:** Faculty of Engineering & Built Environment, National University of Malaysia, Selangor, 43600 UKM, Bangi, Malaysia; E-Mails: anaarshad@yahoo.com (N.S.M.Z.); herdi.leuveano05@gmail.com (R.A.C.L.); jaharah@eng.ukm.my (J.A.G.); faizal.mahmood@ukm.edu.my (W.M.F.W.M.)

**Keywords:** solder paste, fine-pitch component, dynamic characteristic, thermal shock, Taguchi method, solder joint

## Abstract

The significant increase in metal costs has forced the electronics industry to provide new materials and methods to reduce costs, while maintaining customers’ high-quality expectations. This paper considers the problem of most electronic industries in reducing costly materials, by introducing a solder paste with alloy composition tin 98.3%, silver 0.3%, and copper 0.7%, used for the construction of the surface mount fine-pitch component on a Printing Wiring Board (PWB). The reliability of the solder joint between electronic components and PWB is evaluated through the dynamic characteristic test, thermal shock test, and Taguchi method after the printing process. After experimenting with the dynamic characteristic test and thermal shock test with 20 boards, the solder paste was still able to provide a high-quality solder joint. In particular, the Taguchi method is used to determine the optimal control parameters and noise factors of the Solder Printer (SP) machine, that affects solder volume and solder height. The control parameters include table separation distance, squeegee speed, squeegee pressure, and table speed of the SP machine. The result shows that the most significant parameter for the solder volume is squeegee pressure (2.0 mm), and the solder height is the table speed of the SP machine (2.5 mm/s).

## 1. Introduction

In the electronics industries, the manufacture of all electronic components, semiconductors, and computer chips uses Printed Wiring Boards (PWBs) as the base for all of them. In order to pack all electronic components on to PWBs, Surface Mount Technology (SMT) has been required as an assembly technique to form high-quality functional PWBs [1]. Therefore, as an irreplaceable part of many electronic products, the reliability and quality of PWBs need to be considered, to prevent any electrical shorts or unreliable solder joints. Imperfect manufacturing quality of functional PWBs is generally detected on the surface mount. Industry reports and the literature [2,3,4,5,6] indicate that the solder paste printing process causes 50%–70% of the surface mount PWB manufacturing flaws, owing to the reflow and waste of the materials [7]. Since the number of failed mount surfaces increases on PWBs then manufacturing costs also increase. Thereby, fitting an interconnection between electronic components and PWBs requires a sufficient amount of paste, and the variation in the solder volume and solder height should be measured [8].

Previous researchers reported that printing quality performance is greatly influenced by different solder paste materials, PWB designs, stencil aperture designs, and printing process parameters [9,10,11]. Other factors investigated by Pan *et al.* [12] are related to the effect of stencil thickness, solder type, aperture size, aperture shape, and print speed. A robust assembly process for lead-free components used in memory models was developed by Lyer *et al.* [10]. They studied the parameters of the printing process, including solder paste printing, component placement, stencils, PWB land pattern designs, and reflow soldering processes. A systematic approach in the solder printing process was introduced by Ladani *et al.* [11], who investigated the effect of stencil thickness and waiting time before printing, on the creation of the defect. Based on the discussions of prior researchers, we identified the effect of some parameters on the quality of PWBs. This study investigates the effect of solder paste materials and printing processes on PWBs. A solder paste material, usually an alloy or pure metal, is used to integrate electronic components to attachment point in the circuit patterns of a PWB. A solder paste has certain melting point and flow properties to allow distribution by capillary attraction in connecting joint. This paste usually needs silver as a material alloy used for the reflow processing. A paste containing 3%–4% silver achieves the best soldering performance. Adding silver into a solder paste alloy can improve wetting, tensile strength, and creep characteristic. Particularly, silver can significantly influence the wetting time of solder paste when temperature is decreased as it comes into contact with the board. Tensile strength and creep characteristics of the solder paste can be improved by adding silver because of the durability of this element at high temperatures [12].

Nowadays, the significant increase in the cost of metals as the basis of electronic devices has encouraged most electronics industries to provide new materials and methods to reduce the cost of metal [12]. As a result, the price of electronic goods is likely to rise with the increase in the price of metal. On the other hand, customers will still try to look for electronic products that are less expensive but of a high quality. So, this situation is forcing most industries to resolve the problem as quickly as possible, to remain competitive in satisfying customer needs.

To date, most industries have used low-cost base material for electronic devices by changing the content of the solder paste. They also have attempted to introduce various types of solder paste with specific alloy as an alternative solution for the electronics industry to reduce manufacturing costs. However, if the durability of the solder paste content is poor in maintaining a melting point, the solder joint will crack between fine-pitch components and the PWB. Moreover, the high melting point of the solder paste presents a risk in the reflow process and of thermal fatigue failure of the solder joint during SMT production [13]. This characteristic of the solder paste should be properly managed in the mounting process. Hence, this paper studies solder paste reliability after conducting a solder printing process of a fine-pitch electronic component on a PWB.

The evaluation processes will also be conducted in this study to ensure the solder paste is feasible to use and can make a functional PWB. This study primarily aims to examine the reliability of a solder paste alloy. The performance of the solder paste is evaluated after conducting the SMT process. This study performs several tasks to achieve the aforementioned aim. These tasks include evaluating the solder paste condition or solder joint after printing it on the PWB surface; evaluating the appropriate thermal cycle, which can be implemented for the fine-pitch components; and optimizing the significant input parameters on the solder printing process by analyzing the solder volume and solder height.

The remainder of this paper is presented as follows. Section 2 describes the material and methods to examine the reliability of the solder paste alloy. This section particularly explains the mechanism tool for conducting the experiment and the method for testing the solder paste alloy, which are implemented simultaneously during the experiment. Section 3 presents the results of all material and method experiments on the solder paste. It also discusses the solution for the parameters of the solder printer (SP) machine that affects the proper solder volume and solder height. Finally, Section 4 summarizes the result.

## 2. Material and Methods

An electronic manufacturing company in Malaysia that produces electronic devices was selected as the unit of analysis for this case study. This company has made a breakthrough in meeting the market requirement by achieving low material costs. A solder paste alloy that contains 98.3% tin, 0.3% silver, and 0.7% copper has been introduced by this company and is available in the market (*i.e.*, M40-LS720-SX-T4). The solder paste was evaluated using the SMT process as the mechanism of attaching fine-pitch components to the PWB. The solder melting temperature is approximately 220 °C, but the solder paste starts to solidify at 211 °C. A functional PWB is made via nine steps in the SMT process and includes the following:

1. Loader: This process loads the PWB onto the conveyor.
2. Solder Printer (SP) machine: PWB is printed with solder paste by squeegee.
3. Solder paste checker: Checks cooper pad opening is fully covered by the solder paste.
4. Chip shooter: This machine mounts all chip components on to PWB.
5. IC mounter: This machine mounts Integrated Circuit (IC) component on to PWB.
6. Odd shape mounter: This machine mounts odd shape fine-pitch component on to PWB.
7. Reflow oven: Creates a permanent joint between electronic components and their contact pad.
8. Automated Optical Inspection (AOI): This process checks the abnormality of the mounted part.
9. Unloader: The final process of SMT.

The experiment took approximately six months, starting in April 2013 and ending in October 2013. There are three particular method tests to analyze the reliability of solder paste alloy, and include dynamic characteristic tests, thermal shock test, and the Taguchi method. It is important to note that the dynamic characteristic and thermal shock tests consist of several groups of tests that are listed in Table 1 and Table 2, respectively.

**Table 1:** Dynamic characteristic tests.

Method test	Method evaluation	Tools	Testing point	Units
Printing test	Measurement data	Sensei Spois SP 1000	A, B, C	mm^3^
Slumping test	Visual inspection	Keyence VH-8000	D	-
Spreading test	Measurement data	-	D	%
Solder Ball test	Visual inspection	Keyence VH-8000	E	-
Micro-Bridging test	Visual inspection	-	F	-
Wettability test	Visual inspection	Keyence VH-8000	A, B, C	-
Viscosity test	Measurement data	Malcom viscometer	-	Pa.S

**Table 2:** Thermal shock tests.

Test	Method evaluation	Tools	Testing point
Soldering check	Visual inspection	Keyence VH-8000	A, B, C, D, E, F
Heat shock and cross-section	Visual inspection	Keyence VH-8000	A, C, D, E

### 2.1. Dynamic Characteristic Test

**Figure 1:**
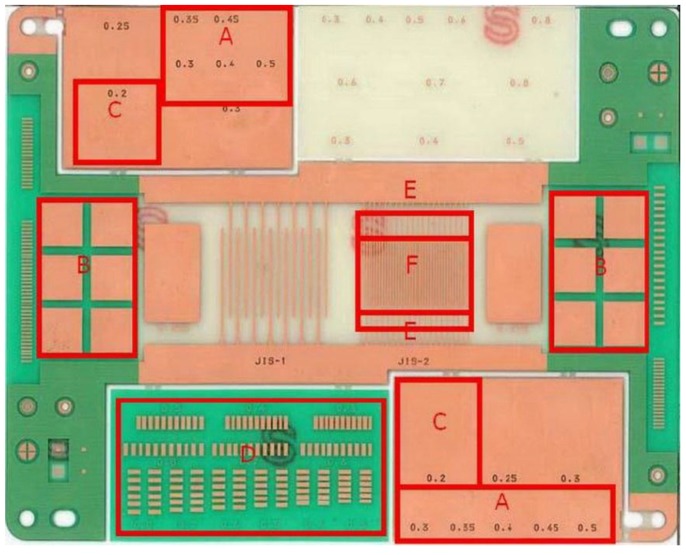
Testing point for the dynamic characteristic tests.

Dynamic characteristic test is used to evaluate the solder paste condition or solder joint after printing it on the PWB surface. This test is usually performed in sequence within five working days. As shown in Table 1, dynamic characteristic consists of seven tests that have different evaluation methods, tools, and specific areas for testing. The testing point or testing zone is shown in Figure 1. A total of six testing areas (A, B, C, D, E, and F) are presented; the different tests on the board depend on the attachment of different types of electronic devices. A brief explanation of this test is as follows:

1. Printing test: This test covers the testing points A, B, and C. A good printing process can be evaluated by measuring the shape, volume, and height of the solder paste that covers the copper pad opening. An acceptable solder shape is described in Section 3.1.1.
2. Slumping test: This test investigates the slumping of solder paste particles after reflow process. Slumping can create a bridge between one dot and another dot of the solder. Slumping test is performed in testing point D. The detailed result of the slumping test is reported in Section 3.1.2.
3. Spreading test: In continuation of the previous test in point D, spreading test is conducted to investigate the spreading rate above 70% to test the hold of the part during the shrinkage of the solder during the melting process. An example of the spreading test is shown in Section 3.1.3.
4. Solder ball test: In point E, the solder ball test is performed to check the ability of the solder paste particle to drag toward the copper pad opening and ensure the number of solder balls at the designed location. Refer to Section 3.1.4 for the result of the solder ball test.
5. Micro-bridging test: This test checks an abnormal appearance, such as a scratch or a fiber, in point F. A video scope is used to observe this abnormality, and the result is presented in Section 3.1.5.
6. Wettability test: Similar to the printing test, wettability test is performed in points A, B, and C. This test ensures that no cold solder occurs during the solder melting process. If wettability is observed on the PWB surface, a low-strength solder joint can occur. An example of the wettability test is discussed in Section 3.1.6.
7. Viscosity test: This test checks the strength and height of the solder. Solder bridging and clogging upon the stencil film opening occurs if the solder is too strong and thick. Therefore, viscosity test is also required to evaluate the stability of the printing process in producing a good solder. The result of this test is shown in Section 3.1.7.

To run this experiment, this study tried to examine 20 test boards. In addition, these boards also contains several fine-pitch components that make electronic devices work.

### 2.2. Thermal Shock Test

A thermal shock test is used to determine the ability of fine-pitch components to bear a sudden alteration of temperature. This alteration is investigated by observing parts exposed to extremes of high and low temperatures. In this case, if there is a coefficient difference of thermal expansion for material composing parts, then cracks could occur. There are two tests for thermal shock, as presented in Table 2. Similar to the dynamic characteristics tests, the testing point or testing zone is shown in Figure 1. These tests are described as follows:

1. Soldering check: This test focuses on the solder appearance, and identifies the kinds of defect, including solder bridging, solder shifting, and solder ball. Soldering check is performed in points A, B, C, D, E, and F.
2. Heat shock and cross-section: Identifies solder-joint condition after testing with a heat shock or thermo-cycling process, within a temperature range of −25 ± 5 °C to 125 ± 5 °C. This test covers the testing points A, C, D, and E.

### 2.3. Taguchi Method

**Figure 2:**
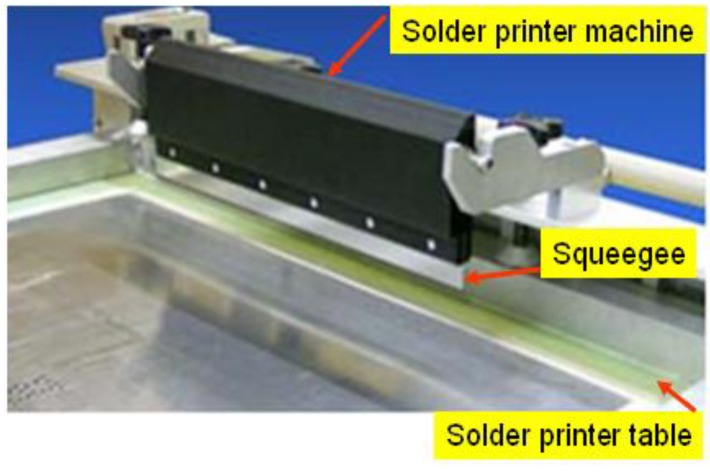
Solder printer machine.

The variation in the solder volume and solder height can critically influence the solder joint [8]. An unreliable solder joint is also caused by difficulties in setting the control parameters of the SP machine. The SP machine is shown in Figure 2.

The control parameters consist of table separation distance, squeegee speed, squeegee pressure, and table speed of the SP machine. The design of the control parameters is based on the Taguchi method to optimize the properties of the solder volume and solder height. This third objective can be accomplished by Taguchi’s approach [14], which provides a systematic and efficient method to determine the near-optimum design parameter for performance.

Taguchi approach performs a robust measure to classify the control parameters that minimize the variability in a process by reducing the effect of uncontrollable factors (noise factors). In this case, a control parameter can be controlled during the experiment and production, whereas the uncontrollable factor can only be controlled during the experiment. Control parameters can be optimized to make a robust process or performance, and even noise factor can be manipulated to reduce the variability to occur using the Taguchi approach. In the Taguchi approach, the noise factor is indicated by the signal-to-noise (S/N) ratio. The value of the S/N ratio depends on the goal of the experiment, which attempts to maximize the S/N ratio for the respective process.

This Taguchi approach has been proved by several studies in successfully optimizing parameters that can affect the machine and tests result [15,16]. Since this experiment considers four control parameters and noise factors with three levels, Orthogonal Array (OA) *L_9_* (Table 3) can be chosen for a minimum number of experiments.

**Table 3:** Standard *L_9_* orthogonal array.

Run	Controllable parameter			
	Table separation distance	Squeegee speed	Squeegee pressure	Table speed of solder printer
1	1	1	1	1
2	1	2	2	2
3	1	3	3	3
4	2	1	2	3
5	2	2	3	1
6	2	3	1	2
7	3	1	3	2
8	3	2	1	3
9	3	3	2	1

For the preliminary test, the range was set (level 1, level 2 and level 3) for each parameter using a measurement. Level 1 and level 3 are considered as upper and lower specification values, while level 2 stands as the middle value. In order to conduct an experiment and assign the column of OA (Table 3), the values of the machining control parameters and noise factors and their levels are presented in Table 4.

**Table 4:** Parameter and noise factors of solder printer machine and their levels.

Controllable parameter	Level 1	Level 2	Level 3
(A) Table separation distance	1.0 mm	1.5 mm	2.0 mm
(B) Squeegee speed	40 mm/s	45 mm/s	50 mm/s
(C) Squeegee pressure	0.100 Pa	0.125 Pa	0.150 Pa
(D) Table speed of solder printer machine	1.5 mm/s	2.0 mm/s	2.5 mm/s

## 3. Results and Discussion

This section emphasizes the evaluation of a solder paste from an enormous number of bonding pads, produced using the SMT process. This section evaluates the reliability of the proposed solder paste material using dynamic characteristic test, thermal shock test, and the Taguchi method. For the dynamic characteristic test and thermal shock test, the experiment is conducted during a mass-production running model. However, for the Taguchi method, the parameter of the SP machine varies for the purpose of studying the best parameter for the solder printing.

### 3.1. Results of Dynamic Characteristic Test

For the first part of the experiment, the dynamic characteristic test was done according to the characteristics or features of the solder paste, which might vary according to the base material of the solder. The experiments required 20 pieces of PWB. The result of this test is shown by several characteristic tests, including:

#### 3.1.1. Printing Test

**Figure 3:**
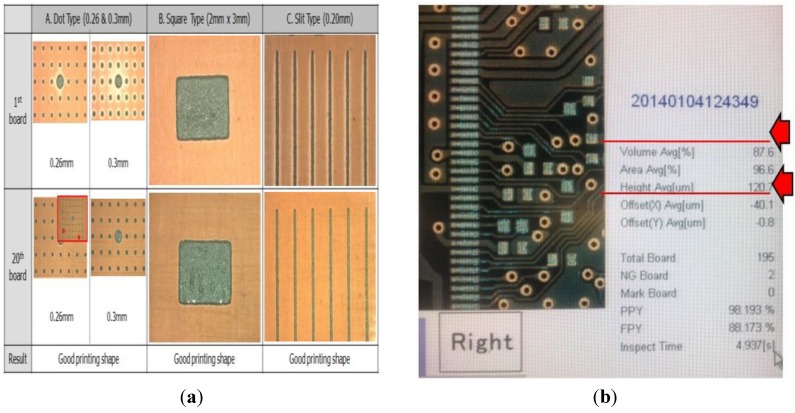
(**a**) Acceptable shape of the solder for all 20 pieces of the board; (**b**) acceptable volume and height of the solder for printing tests.

The printing test evaluates shape, volume, and height of the solder that was attached to the PWB. Using visual inspection, the shape of solder falls into three types: dot, square, and slit. To be acceptable a dot shape must have a diameter of 0.25 mm–0.5 mm; the square type area must be 2 mm × 3 mm; and the length of the slit type must be 0.2 mm, as shown in Figure 3a. The size of the particles dispersed in the solder paste ranges from 25 µm to 36 µm (sphere).

The solder volume and solder height are measured using a solder paste inspection machine. As presented in Figure 3b, the machine can detect the volume and height precisely. On the machine reader, the solder volume must be more than 80%, while the value solder height is 115 µm or more. 

#### 3.1.2. Slumping Test

**Figure 4:**
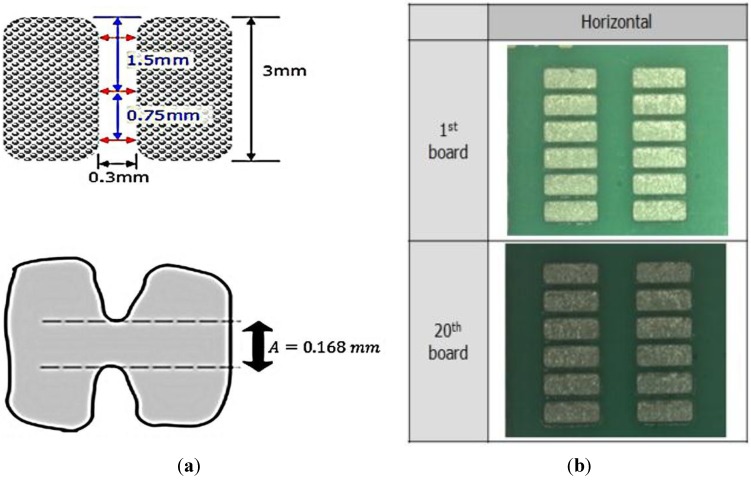
(**a**) Calculation of the bridges and the gap rate; (**b**) The result of the slumping tests for all 20 board pieces.

This test investigates any slumping of the melting solder that can cause a bridge. As shown in Figure 4a, a bridge between one solder and another solder cannot more than 1 mm, and a gap rate *G* (%) should be 50% with tolerance ±10%. Also, refer to Figure 4a, a gap rate can be calculated as *G* (%) = Remaining Gap (*A* = 0.168 mm)/(0.3 mm × 100), hence *G* (%) = 56%. In addition, the results of 20 pieces of board are shown in Figure 4b. All results shows that there are no slumping of the melting solder that can cause a bridge.

#### 3.1.3. Spreading Test

**Figure 5:**
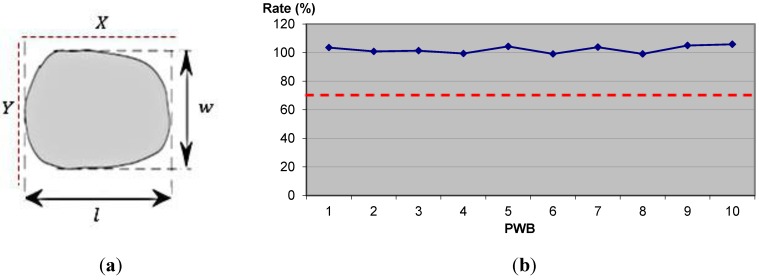
(**a**) Calculation of spreading test; (**b**) example of the spreading rate for 10 board pieces.

The spreading test evaluates the spread rate of the solder on the PWB, and serves to suppress failure caused by insufficient spread of the solder. A good spreading condition is a spreading rate over 70%. An example of the spreading test is shown in Figure 5. As shown in Figure 5a, in order to calculate the spreading test, given length (*l*) = 2.0 mm, width (*w*) = 0.5 mm, printing area (*X* × *Y*) = 2.5 mm × 0.4 mm, and spreading rate (%) = (2.0 mm × 0.5 mm)/(2.5 mm × 0.4 mm) × 100%. Based on Figure 5b, taking the example of 10 pieces boards, all results show that, on average, the spreading rate is 100%.

#### 3.1.4. Solder Ball Test

**Figure 6:**
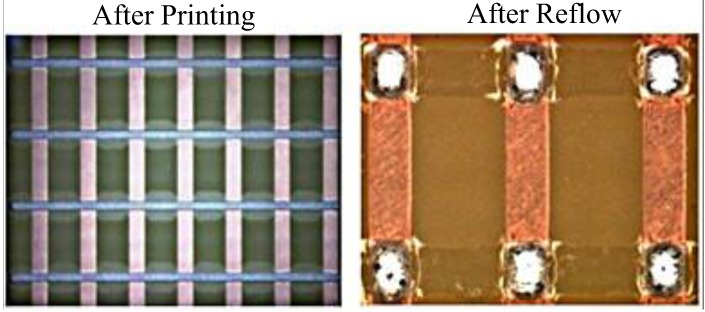
Results of solder ball test.

This test considers an evaluation item to prevent electric short circuit failures, owing to the frequent occurrence of, solder balls, with a magnification of 40–50 times using an optical microscope. Figure 6 presents examples of the solder ball tests. Moreover, it shows that there is no solder ball in the printing process.

#### 3.1.5. Micro-Bridging Test

**Figure 7:**
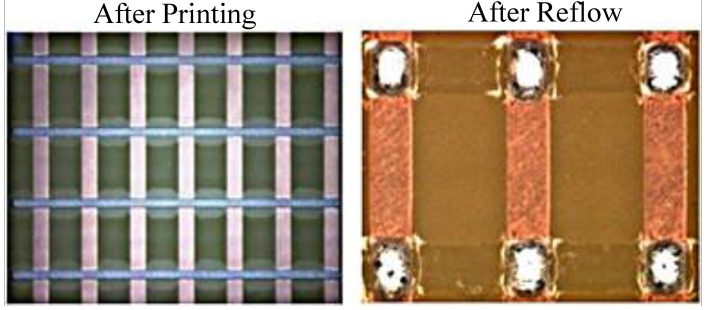
Results of micro-bridging test.

The micro-bridging test is an evaluation item to prevent short circuit failures as a result of micro-bridges. The method is used to check whether there a micro-bridge happens in Area F of the test board. Referring to Figure 7, after the printing process and reflow process, there is no micro-bridge in any of the PWBs.

#### 3.1.6. Wettability Test

**Figure 8:**
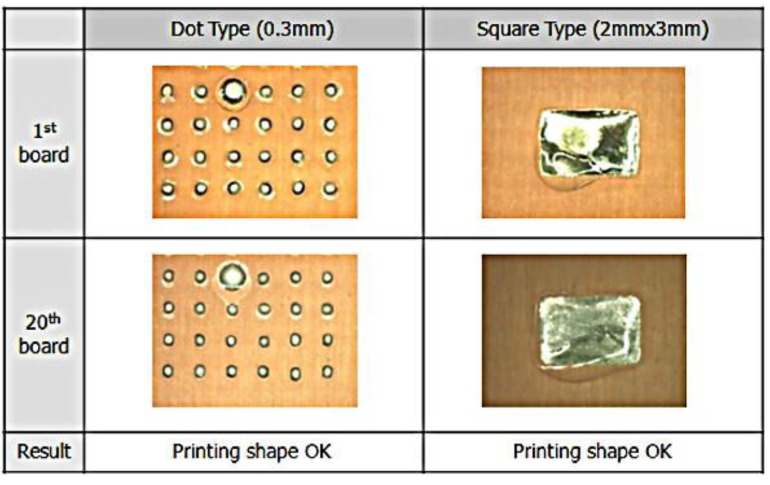
The wetting condition of the dot and square type solder shapes.

This test checks the wetting solder condition of the liquid flux. After conducting experiments for all 20 pieces of the PWB, as shown in Figure 8, there is no wettability for the dot and square type solder shapes. It can be inferred that if there is no wettability occurring then electronic components and PWB have strong solder joints.

#### 3.1.7. Viscosity Test

**Figure 9:**
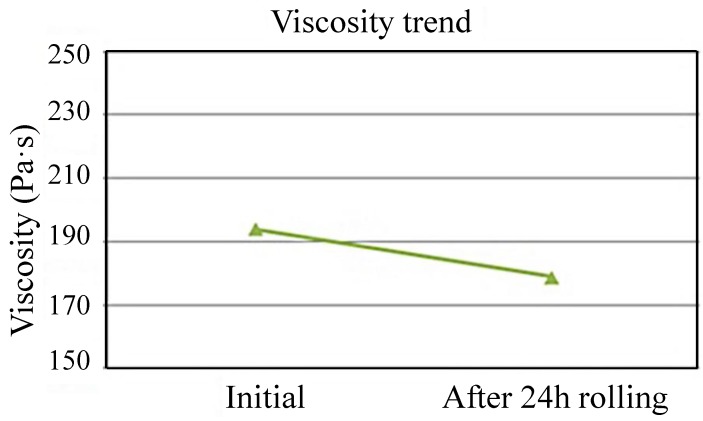
Examples of viscosity test.

The viscosity test is to maintain the stability related to the total printability of the solder paste. This test differs from other dynamic characteristic tests, as there is no board involvement in this test. The solder paste is examined using a viscometer machine, where the temperature of the machine is set at 25 °C. A good solder paste should be in the range between 160 Pa·S and 200 Pa·S. The result of this experiment is shown in Figure 9.

### 3.2. Result of Thermal Shock Test

The thermal shock test has long been used as an industry standard to evaluate a solder joint’s reliability in electrical products. The reliability of the solder joint is a function of the heat coefficient of the material expansion. This heat coefficient can be evaluated when there is a sudden alteration of temperature. This alteration can cause a thermal gradient of the material causing stress that exceeds the strength of the material, so that a crack will happen to the solder joint. Therefore, this coefficient is referred to as the difference between stress and strength of the material that can be indicated by (Δ*T*).

**Figure 10:**
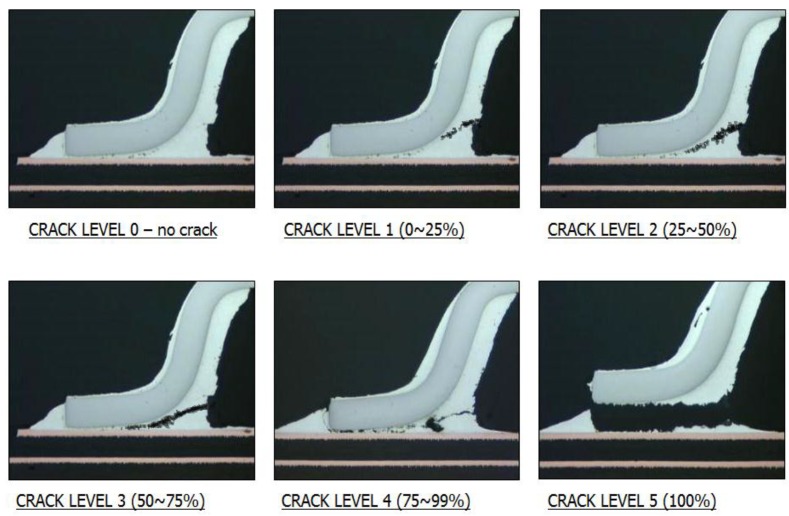
Crack levels of the solder joint.

Experiments are carried out with the soldering check, heat shock, and cross-section. For the soldering check, there are five levels, as shown in Figure 10, where level 0 represents no crack, while levels 1–5 are categorized as crack with tolerance 0%–25%, 25%–50%, 50%–75%, 75%–99%, and 100%, respectively. In this case, level 5 is considered an unacceptable solder with totally defective boards. Several works and repairs are required to restore the solder to its original quality. All these crack levels can be investigated using visual inspection and possibly occur to all fine-pitch components.

However, to identify which crack level happens to the solder joint, the heat shock test is then performed. The first step of this test is the thermal cycle (TC) calculation for each fine-pitch component. There are seven components to be tested that already join on to the PWB. Initially, this heat shock test can be done by increasing the temperature of the heat shock machine when the power is “on” and permitting a normal temperature decrease when the power is “off”. The results of this test can be seen in Table 5.

**Table 5:** Thermal cycle calculation.

Components	Part 1	Part 2	Part 3	Part 4	Part 5	Part 6	Part 7
Power supply ON (°C)	88.5	83.2	66.1	79.8	52.9	52.1	67.3
Power supply OFF (°C)	45.5	89.0	45.5	45.5	45.5	45.5	45.5
ΔT (°C)	43.0	5.8	20.6	34.3	7.4	6.6	21.8
Days	20	11	4	12	1	1	4
Actual test × 1.5 safety factor	1446	779	256	835	28	22	291
Test requirement (Cycle)	1446	779	291	835	28	28	291

Each fine-pitch component that is joined to the PWB was put into chamber and taken out when the thermal cycle was complete, as shown in Table 5. The table shows that each component has a different treatment to analyse its quality when the alteration of temperature occurs within the range of −25 ± 5 °C to 125 ± 5 °C. As the result, this experiment causes the expansion of mechanical stresses that depend on the thermal and mechanical characteristics of the components and vary with time, owing to changes in the temperature distribution through the component, which stimulate the solder crack. This solder crack then is categorized into its crack level. Based on this experiment, there are three parts, Part 1, 2 and 7 categorised in level 1, which can be considered as a minor crack. However, this minor crack is still reliable for the quality of electronic circuits.

### 3.3. Results of Taguchi Method

In this section, the results of the orthogonal array Taguchi method for determining optimal parameters of SP machine are reported, which are used to print out the solder paste onto the PWB. Using *L_9_* orthogonal array, the experimental combination of SP machine and signal to noise ratio for each performance is presented in Table 6. The S/N ratio measures the variable response relative to the nominal or target value under different noise conditions. The response to noise factor was minimized to achieve the goal of the experiment.

**Table 6:** *L_9_* Taguchi experimental design and S/N ratio of solder volume and solder height.

Experiment	A	B	C	D	(S/N)	
					Solder volume	Solder height
1	1	1	1	1	28.926	24.067
2	1	2	2	2	36.711	32.227
3	1	3	3	3	40.375	48.282
4	2	1	2	3	32.778	36.565
5	2	2	3	1	28.393	36.686
6	2	3	1	2	47.477	36.948
7	3	1	3	2	44.082	34.872
8	3	2	1	3	36.900	44.179
9	3	3	2	1	44.032	41.419

Note: A: Table separation distance, B = Squeegee speed, C = Squeegee pressure, D = Table speed of solder printer.

As mentioned in the previous Section 2.3, the solder volume and solder height are the two responses or quality performances considered in this study. However, the parameter data (table separation distance, squeegee speed, squeegee pressure, and table speed of SP machine) that influence the quality performances are shown in Table 7.

**Table 7:** Data obtained from experiment using Minitab 16.

A	B	C	D	Trial V1	Trial V2	Trial V3	Trial H1	Trial H2	Trial H3
1.0	40	0.100	1.5	112.7	121.0	117.20	132.0	117.5	130.3
1.0	45	0.125	2.0	112.6	109.5	111.33	114.9	118.3	112.7
1.0	50	0.150	2.5	108.9	110.1	110.00	113.6	112.8	112.9
1.5	40	0.125	2.5	111.3	114.3	111.60	112.8	114.6	116.2
1.5	45	0.150	1.5	113.9	106.7	111.60	116.3	115.9	113.2
1.5	50	0.100	2.0	112.3	111.6	111.77	114.8	117.0	113.8
2.0	40	0.150	2.0	106.1	106.2	106.53	117.0	115.8	113.6
2.0	45	0.100	2.5	114.6	113.4	113.13	112.9	113.7	114.3
2.0	50	0.125	1.5	112.4	111.0	111.73	114.6	115.2	113.3

Note: A: Table separation distance, B = Squeegee speed, C = Squeegee pressure, D = Table speed of solder printer.

**Figure 11:**
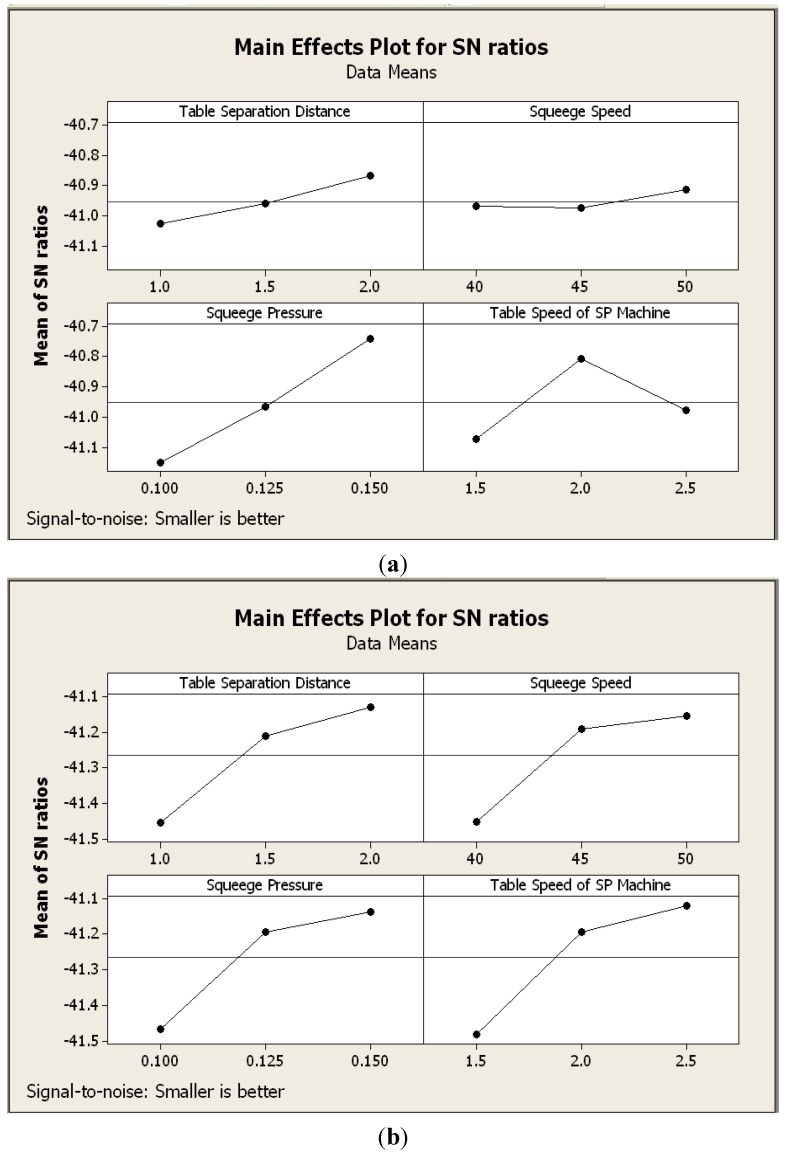
(**a**) The effect of solder printer machine parameter on solder volume; (**b**) The effect of solder printer machine parameter on solder height.

In the Taguchi method, the quality response to optimize the parameter of SP machine needs to be considered first. In this case, a smaller S/N shows the most significant characteristic of optimizing the SP machine for both solder volume and solder height. To conduct this experiment, MINITAB 16 was used. The results of investigating the parameter of SP machine to quality performance (solder volume and solder height) are shown in Figure 11.

Based on Figure 11, the recommended parametric combination for optimum solder paste volume is the table separation distance (2.0 mm), squeegee speed (50 mm/s), squeegee pressure (0.15 Pa) and table speed of SP machine (2.0 mm/s). In the case of solder paste height, the optimum recommended parametric combination is the table separation distance (2.0 mm), squeegee speed (50 mm/s), squeegee pressure (0.15 Pa) and table speed of the SP machine (2.5 mm/s). The results also show that the solder paste volume settings are mainly the squeegee pressure, followed by the table speed of the SP machine, while, for the solder height, parameters are mainly contributed by the table speed of the SP machine, followed by squeegee pressure. It means that squeegee pressure and the table speed of the SP machine are the most important factors, and significantly affect the volume and height of the solder paste.

## 4. Conclusions

In this paper, the reliability of a solder paste alloy (tin 98.3%, silver 0.3%, and 0.7%) is investigated for construction of the surface mount fine-pitch component on a PWB. The implication of this study is to ensure the quality of a solder alloy material as the electronic industries continue to advance, by providing a low-cost but high-quality material. In addition, this alloy has become increasingly popular where it can mitigate a drop in electric shock performance.

Based on the conducted experiment, the first test (dynamic characteristic tests) aimed to analyse 20 boards by investigating the solder joint. During this dynamic characteristic test, all of the evaluations, including printing test, slumping test, spreading test, solder ball test, micro-bridging test, wettability test, and viscosity test, fulfilled the specification of acceptable quality for the solder joint. Meanwhile, in the second test, the thermal shock test was used to test the reliability of the solder joint by putting the board into a chamber with an alteration of temperature. This situation caused the expansion of thermal material which stimulated the solder to crack. The test result shows that the crack of the solder joint lies on crack level 1, which is categorized as minor crack. However, this condition is still an acceptable and reliable solder joint.

Moreover, the Taguchi method can determine the optimal parameter of the SP machine that influences the solder volume and solder height. The most significant parameter that influences the solder volume is squeegee pressure, while solder height is influenced by table speed. Thereby, all the experimental results clearly show that the product still fulfils the specification of solder paste quality. This solder paste alloy can, therefore, be introduced as a competitive product in order to provide a low-cost material.

For future research, some possible extensions could be considered. Rapid technology development has led to the delivery of various new SMT components in the market. Thus, it is an advantage to study various types of copper points such as square type and double opening footprint that effect the solder joint. The mixing of solder paste with flux has also attained the best possible bonding between solder and metal. This is because flux can enable the solder mass to wet the surfaces that are to be joined. Therefore, the study of several types of fluxes is useful in examining the effect on solder joint and strength.
